# Non-Intrusive Load Identification Based on Retrainable Siamese Network

**DOI:** 10.3390/s24082562

**Published:** 2024-04-17

**Authors:** Lingxia Lu, Ju-Song Kang, Fanju Meng, Miao Yu

**Affiliations:** College of Electrical Engineering, Zhejiang University, Hangzhou 310027, China; lulingxia@zju.edu.cn (L.L.); 3170300663@zju.edu.cn (J.-S.K.); fanju_meng@zju.edu.cn (F.M.)

**Keywords:** non-intrusive load monitoring, V-I trajectory, Siamese network, embedded Linux system

## Abstract

Non-intrusive load monitoring (NILM) can identify each electrical load and its operating state in a household by using the voltage and current data measured at a single point on the bus, thereby behaving as a key technology for smart grid construction and effective energy consumption. The existing NILM methods mainly focus on the identification of pre-trained loads, which can achieve high identification accuracy and satisfying outcomes. However, unknown load identification is rarely involved among those methods and the scalability of NILM is still a crucial problem at the current stage. In light of this, we have proposed a non-intrusive load identification method based on a Siamese network, which can be retrained after the detection of an unknown load to increase the identification accuracy for unknown loads. The proposed Siamese network comprises a fixed convolutional neural network (CNN) and two retrainable back propagation (BP) networks. When an unknown load is detected, the low-dimensional features of its voltage–current (V-I) trajectory are extracted by using the fixed CNN model, and the BP networks are retrained online. The finetuning of BP network parameters through retraining can improve the representation ability of the network model; thus, a high accuracy of unknown load identification can be achieved by updating the Siamese network in real time. The public WHITED and PLAID datasets are used for the validation of the proposed method. Finally, the practicality and scalability of the method are demonstrated using a real-house environment test to prove the ability of online retraining on an embedded Linux system with STM32MP1 as the core.

## 1. Introduction

Nowadays, the progress and development of advanced metering infrastructures can provide strong technical support for the efficient management of electrical energy consumption [1,2]. As one of the key techniques for energy management, load monitoring can provide detailed usage information for the consumers to better control their electricity consumption behaviors and, ultimately, save energy [3,4]. With the aid of load monitoring, the consumers can save up to 15% of energy [5].

Load monitoring, in general, can be divided into two categories, namely, intrusive load monitoring (ILM) and non-intrusive load monitoring (NILM). ILM is based on hardware, which requires the installation of a monitoring device for each load. Thus, this method analyzes the load consumption with a high precision. However, it is not cost-effective due to the complex hardware installation, which has a high cost. NILM, a concept originally developed by Hart [6], focuses on an algorithm and only needs to deploy a meter at the entrance of each household. Compared with ILM, NILM has a lower cost in terms of a lower amount of hardware deployment, easier maintenance, and increased protection of user’s privacy [7]. Hence, NILM has much broader application prospects than ILM.

NILM has not received adequate attention, after being proposed in the initial stage, due to its high computation requirements, complex algorithm, and low accuracy. However, with significant improvements in computer technology and artificial intelligence, the previous issues associated with NILM can be solved by the emerging technologies and algorithms; therefore, more researchers have switched their focus to this field.

As an essential step of NILM, load feature extraction is a core concept in this method. At the early stages, active and reactive power are two major indicators and they are combined with traditional machine learning methodologies for load identification. For instance, in [8], the load switching event is detected by comparing the power changes to a user-set threshold, and the load is identified by using a complex power signature based on the magnitude of active and reactive power. Although this algorithm has nearly no requirements in computation and implementation, the growing electrical loads will greatly influence the identification accuracy.

In recent decades, NILM has benefited from advances in sensors innovation and the widely applied technologies in signal analysis, which could implement the higher-dimensional load feature identification through machine learning algorithms. For instance, the active power, reactive power, current, and power factor are used as the inputs of a multi-objective classification algorithm based on random k-label sets for load identification [9]. Qureshi et al. have introduced an event-based non-intrusive disaggregation algorithm that uses Gaussian mixture models (GMMs). This algorithm can automatically detect appliances with two states by analyzing aggregated data, while applying the Bayesian information criteria (BIC) to identify the best number of clusters [1]. Additionally, Luan and Yang disaggregate the aggregated power data into individual load power data by using the Hidden Markov Model (HMM) [10], which is suitable for the later analysis of electricity consumption data. Then, Wang et al. introduce a new Adaptive Factorial Hidden Markov Model (Adaptive-FHMM) to track the changing working states of individual appliances, which features an adaptive clustering mechanism that autonomously adjusts the number of hidden states in response to changes in power usage during different operational phases and leverages this detailed state information from each appliance to create an integrated model to forecast the power consumption of the appliance [5]. However, both methods have a huge computational burden. In [11], the load features are obtained using wavelet analysis and then the machine learning algorithms, such as decision tree and semi-supervised machine learning, are used to identify the load. Deep learning techniques are widely used in feature extraction and in the load identification process. In [12], a temporal convolutional neural network is used to automatically extract high-level load signatures for individual appliances. In [13], the deep convolutional neural network is utilized to provide a solution for load disaggregation.

In addition, the sequence-to-point (seq2point) learning and sequence-to-sequence (seq2seq) learning algorithms are commonly used for energy disaggregation. Seq2point learning is first proposed with a CNN in [14] as a method of NILM, where the input is a window of the total energy data and the output is a single point of the target appliance. Further, different algorithms are incorporated into seq2point models for energy disaggregation to improve the identification accuracy, for example, the temporal convolutional network (TCN) [15], the bidirectional dilated residual network [4], the discrete wavelet transform [16], and the bi-directional TCN [17]. Different from the seq2point model, the seq2seq-based NILM aims to output the sequences, of an equal length to the input, that contain only the power consumption of a single appliance. In [18,19], LSTM and an autoencoder combined with an attention mechanism are employed for seq2seq load aggregation models, respectively. These approaches rely on calculation and processing using intelligent algorithms; thus, they generally require a large amount of computation resources.

Due to the rich information represented by the V-I trajectory and the significant progress in computer graphic processing, a V-I trajectory image has been incorporated into NILM as a crucial load feature and the load identification accuracy can be prompted by deploying the deep neural network. In [20], a V-I trajectory extraction approach is proposed based on the steady-state data before and after an event and a support vector machine multi-classification method is applied for load identification. In [21], a weighted pixelated image of the V-I trajectory is utilized as the CNN input to enhance the extraction process of crucial features and the method is tested on both PLAID [22] and WHITED [23] datasets. In [24], the reconstructed image of the V-I trajectory is used as the input for the CNN to classify the appliances, and the identification accuracy of similar appliances can be improved. Liu and Wang have previously employed the transfer learning method with the AlexNet model for NILM [3]. With the trajectory preprocessing and color encoding techniques, different electrical loads can be distinguished by using the shape and color information of their V-I trajectories and a high identification accuracy can be achieved. A large number of new methods from the combination of different feature selection techniques with several classification algorithms are investigated in [25] and several NILM methods using the V-I trajectory features are overviewed in [26].

Although the aforementioned methods can achieve an ideal identification accuracy in theoretical calculations, they are still challenging to implement in a practical situation. There are two main concerns. Firstly, these methods cannot provide a reliable unknown load identification solution. Classical load identification methods rely heavily on historical data to train the classification model, which is incapable of identifying new unknown loads. Secondly, the high-level identification accuracy depends on the precise training model, which requires large-scale training data [27]. But the load database is not easy to obtain in practice.

In recent years, a growing number of methods have been proposed for unknown load detection. A new appliance detection method is proposed for NILM using the features of switching ON or OFF [28]. Yin et al. suggest a method for identifying unknown loads by using a low-dimensional feature space from Siamese neural networks to determine the feature similarities and incorporate transfer learning to construct the pre-identification model for unknown loads by facilitating category-added learning [29]. The first step is pre-classification using a one-dimensional CNN model and, in the second step, according to the classification results, the similarity of the space convex hull overlap rate with the Siamese neural networks is calculated. In addition, NILM algorithms based on Siamese networks have been proposed by Baets [30] and Yu [31], respectively. These methods can accurately detect the unknown load using the V-I trajectory representation, but still cannot satisfy the practical situation. The binary V-I trajectory feature used for load identification in [30] is not distinctive enough to recognize appliances that fall into the same category, because it cannot reflect the power features [32]. Another problem is that, due to the newly discovered unknown load data that did not participate in the training of the identification model, the identification accuracy for newly discovered loads is not high [31]. Kang [33] has proposed an adaptive NILM method using an autoencoder network and a TOPSIS algorithm. Two feature vectors are obtained using Fast Fourier Transform (FFT) and an autoencoder model and then the similarity between the load feature vectors is calculated using the TOPSIS algorithm. This method can also accurately detect unknown loads and the current feature is used to make up for the disadvantage of the V-I trajectory that cannot reflect the power features, but the identification accuracy is also influenced by the number of unknown loads. The reason for this is that the identification model is not updated synchronously as new loads are added. Although these models can be retrained to increase the identification accuracy of new loads, the time cost is unacceptable and the high demand for computation resources is also challenging for embedded devices [34].

In light of the above discussions, it is necessary to design a method that can detect the unknown load and maintain a high identification accuracy when the number of unknown loads increases, which is beneficial to implement the method in practical situations.

Therefore, this paper has proposed an approach that can enhance the identification ability of unknown loads by updating the system model in real-time. Firstly, the Siamese networks are used to calculate the similarity of the V-I trajectory features and perform a preliminary classification. It contains the following three subnets: a fixed CNN and two retrainable BP networks. The CNN and one of the BP networks are responsible for data compression of the V-I trajectory, with the aim of obtaining low-dimensional features. The other BP network is focused on calculating the similarity between the V-I trajectory features in the database. In addition, there is a situation where the preliminary classification is invalid. For instance, for the resistive loads with the same internal circuits, their V-I trajectories are highly similar. To distinguish different types of loads with similar V-I trajectories, power feature matching is further implemented. After the detection of unknown loads, these two BP networks will be retrained and upgraded in real-time on the embedded device. The user will be reminded to label the new load when an unknown load is detected and the system can be upgraded online using the subsequent retraining process.

A comparison of the proposed method with several existing typical methods is shown in Table 1 and the main contributions of this paper are as follows:

(1) Compared with the energy disaggregation method in [10,15], the proposed model can detect the unknown load by using feature fusion of the V-I trajectory and the power feature.
(2) Compared with the conventional V-I trajectory-based load identification methods such as those in [30,31], the identification accuracy can be improved by dynamically updating the feature database and retraining the model.
(3) In the model retraining process, only the BP network parameters need to be finetuned and the CNN remains unchanged. Therefore, it can be deployed on the embedded Linux system without PC and Server support.

The remainder of this paper is organized as follows: Section 2 introduces the principle of the proposed load identification method based on the retrainable Siamese network. The following section demonstrates the Siamese network structure and training process. And then, Section 4 aims to validate the performance of the proposed method through public datasets and to compare it with other methods. Moreover, this section proves the feasibility of the online training of the BP networks through experimental results. Finally, Section 5 concludes the methodology’s strengths and indicates its limitations.

## 2. Principle of Load Identification Based on a Retrainable Siamese Network

This section will introduce the principle of the proposed load identification algorithm and load features used in this paper.

### 2.1. Load Identification Process

**Figure 1:**
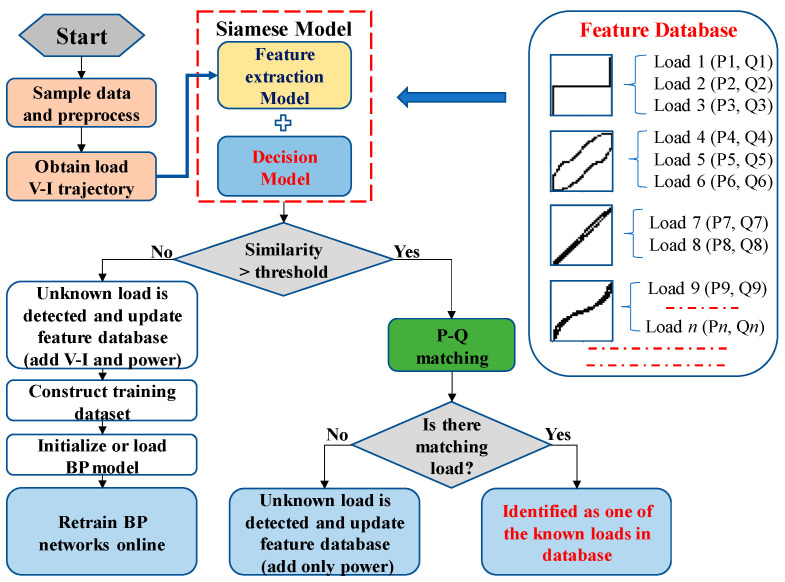
The workflow of the proposed method.

The load identification workflow of the proposed method is shown in Figure 1. Before load identification, it is necessary to pre-train the feature extraction model on a PC or Server. Assuming that only one load is switched on or off at a time, the voltage and current waveform of a single load can be extracted by calculating the steady-state voltage and current difference before and after the switching event.

The load identification process is as follows:

(1) Collect the steady-state voltage and current of the load using a high sampling rate with the minimum number of one cycle of data points;
(2) Normalize the voltage and current and obtain the V-I trajectory image of the load;
(3) Input the V-I trajectory image into the Siamese network to calculate the similarity with the known V-I trajectory in the feature database;
(4) Compare the similarity with the preset threshold for preliminary identification.
  - When the similarity is less than the threshold, it will be recognized as an unknown load. The feature database is updated by adding both the V-I trajectory feature vector and the corresponding power feature. The training set is constructed of pairs of V-I trajectory feature vectors. Then, the two BP networks in the Siamese model are retrained in real-time.
  - When the similarity exceeds the threshold, the power features are further analyzed through the length ratio and cosine distance between the power features. When similar power features exist, the load is identified as one of the known loads. Otherwise, when there are significant differences with the known power features, the load will be marked as new and the feature database is updated by adding only the power feature.

### 2.2. V-I Trajectory Image

The V-I trajectory belongs to the high-frequency features, which can reflect the load features such as harmonic characteristics, the phase angle difference between voltage and current, and electronic appliance conduction characteristics during the steady-state operation [35].

The algorithm to obtain the V-I trajectory image is referred to in [3]. First, the steady-state voltage and current waveforms from appliances that are operating continuously are collected. The voltage and current values are then normalized by dividing their respective maximum absolute values. Next, we segment the plane defined by voltage and current into a 2N × 2N grid of cells and interpolate the original waveform trajectory to maintain the continuity of the curve after converting it into a binary representation form. Finally, these interpolated points are mapped onto the 2N × 2N matrix grid, which has been initialized to all zeros, and the cell is marked as 1 when the data point that the V-I trajectory is mapped to is within the valid range of the grid, otherwise it will be marked as 0. Thus, the image size is the key point in this scenario and the resolution of the V-I trajectory image is set as 32 × 32 in this paper, because the shape features of the V-I trajectory will not be distinctive enough if the pixel number is too small, as it is easy to cause the loss of the high sampling rate data information [24]. On the contrary, if the pixel number is too large, the noise and disturbance in the images will become sharp and will deteriorate subsequent feature extractions [3].

After obtaining the V-I trajectory image based on the voltage and current data, the proposed method inputs the image into the pre-trained Siamese network to calculate the similarity with existing V-I trajectory features, for a preliminary classification.

### 2.3. Power Feature Matching

One V-I trajectory image can correspond to different loads; for instance, the resistive loads with the same internal circuits but different powers. Since the V-I trajectory cannot reflect the load power features, the power feature matching process is further required. The cosine distance and length ratio between the power feature vectors are calculated as follows:

(1) $$cos<\vec{a},\;\vec{b_{i}}>=\frac{PP_{i}+QQ_{i}}{\sqrt{\left(P^{2}+Q^{2})(P_{i}^{2}+Q_{i}^{2}\right)}}$$

(2) $$lr=\frac{min(\left|\vec{a}\right|,\left|\;\vec{b_{i}}\right|)}{\;max(\left|\vec{a}\right|,\left|\;\vec{b_{i}}\right|)}=\frac{min(\sqrt{P^{2}+Q^{2}},\;\sqrt{P_{i}^{2}+Q_{i}^{2}})}{max(\sqrt{P^{2}+Q^{2}},\;\sqrt{P_{i}^{2}+Q_{i}^{2}})}$$

(3) $$sim\left(\vec{a},\;\vec{b_{i}}\right)=0.5*cos<\vec{a},\;\vec{b_{i}}>+0.5*lr$$

(4) $$\vec{a}=(P,\;Q)$$

(5) $$\vec{b_{i}}=(P_{i},Q_{i})$$

where P and Q are the active and reactive power features of the load to be identified and $P_{i}$ and $Q_{i}$ are the active and reactive power features of the loads in the corresponding dataset. $cos<\vec{a},\vec{b_{i}}>$ is the cosine distance that represents the angle between two vectors, lr represents the magnitude relationship, and $sim\left(\vec{a},\vec{b_{i}}\right)$ means the similarity between power features.

The load power features are marked as being in the feature database when the value of (3) exceeds the threshold. Otherwise, these load power features will be labeled as a new load and the feature database will be updated.

## 3. Retrainable Siamese Network

This section will introduce the structure and the training process of the retrainable Siamese network.

### 3.1. Introduction of the Siamese Network

The new load cannot be predicted, as it could appear at any time. The modern NILM system should be able to identify these new loads [28] and improve the model performance through self-updating. However, most of the neural network models for load identification are focused on solving the classification problem. These classification models require numerous training data to support, cannot address the issue of detecting unknown loads, and cannot automatically update the network parameters in real-time.

**Figure 2:**
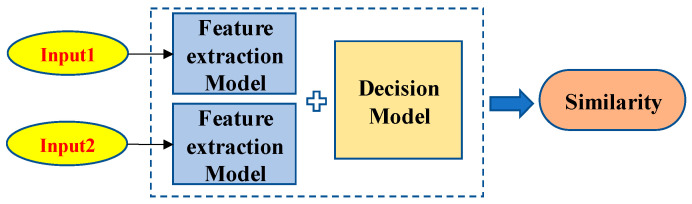
The structure of a Siamese network.

In this paper, the Siamese network is used for the feature extraction and similarity calculation between different V-I trajectories. A Siamese network, as shown in Figure 2, is a special kind of neural network mainly utilized to measure the similarity of two inputs [36]. The two feature extraction models share all the weights, which means that only one network is needed to be trained. The two inputs are passed through the “Feature extraction Model” to obtain new low-dimensional feature vectors and then the similarity between the two inputs is calculated using the “Decision Model”. When the similarity is larger than the preset threshold, it means that these two input V-I trajectories are the same; otherwise, these two input V-I trajectories are different.

Siamese networks are dedicated to addressing two comparable inputs, which have been widely used in numerous fields such as face recognition and fingerprint verification. In [37], a complete low-resolution face recognition system is developed using the Siamese network as part of the facial recognition component. In [38], a Siamese CNN encoding network is constructed to measure the distance of input samples, which significantly reduced the demand of training samples.

Compared with traditional networks, the Siamese network has two significant advantages for NILM. Firstly, it can accurately detect unknown loads. The similarity between the V-I trajectory to be identified and each known V-I trajectory in the database is calculated and is then compared with the threshold value. When the maximum similarity is larger than the threshold, it is considered that the V-I trajectory to be identified is one of the V-I trajectories from known loads and then the power features are further matched. When the maximum similarity is less than the threshold, it is considered that the V-I trajectory to be identified is not in the database, that is, the load is an unknown load and, therefore, the new load needs to be labeled and the network model needs to be retrained. Secondly, the Siamese network can optimize the quantity of the training datasets. Since the network accepts two inputs, samples can be randomly combined in pairs, which can extend the dataset. Thus, it can greatly reduce the number of samples required for training.

### 3.2. Self-Adaption of the Siamese Network

Although the Siamese network has a high-performance in detecting unknown loads, the representation ability of the network will be influenced by the growth in the number of unknown loads. The retraining network model can mitigate this negative impact by rectifying the model parameters when unknown loads are detected. However, the time cost of retraining the whole network is considerably high and it demands a large number of computation resources.

**Figure 3:**
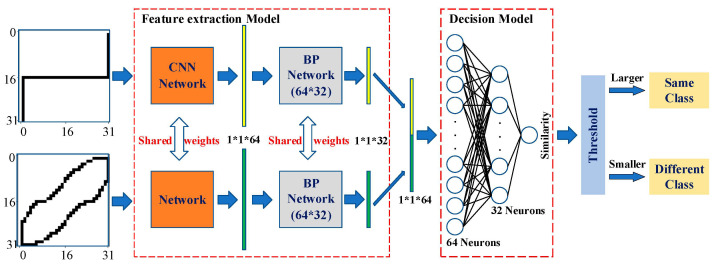
Retrainable Siamese network.

In this paper, the Siamese network is divided into the feature extraction model and the decision model, to achieve the automatic update in real-time, as shown in Figure 3. The feature extraction model consists of a fixed CNN network and a retrainable BP network. The decision model only includes a single retrainable BP network that focuses on calculating the similarity between the feature vectors of the two V-I trajectories.

In the Siamese network, the CNN network for feature extraction is built based on the lightweight Lenet-5 model. Although there are more precise models in image processing, such as the AlexNet model and the VGG-16 model, since the V-I trajectory image is not as complex as the face image and the target of this method is online identification on embedded devices, the structure of the lightweight Lenet-5 model is considered adequate. The structure of the BP network is not complex, which helps to satisfy the lightweight requirements. Compared with other machine learning or deep learning designs, the proposed Siamese network has two advantages. First, it is partially retrained. Since the BP networks have a relatively simple structure, they can be retrained online to fulfill the real-time requirement. Second, the whole structure is lightweight. Since both the Lenet-5 model and BP networks are not complex, the whole network can be implemented on the embedded devices.

Since the CNN network is fixed, the feature vectors extracted using the CNN are saved in the feature database. Therefore, when calculating the similarity between V-I trajectories, only the load V-I trajectory to be identified needs to go through the CNN for feature extraction and this is then compared with the known load feature vectors in the database to calculate the similarity, which can greatly reduce the identification time. Also, this design can reduce the calculation and time costs of training on the embedded system, enhancing the whole network performance.

The feature extraction model and the decision model are trained separately. First, the feature extraction model (including the CNN and the first BP network) is trained. The model inputs are the V-I trajectory images and the outputs are the extracted feature vectors. The loss function is a contrastive loss function defined as follows:

(6) $${loss}_{f}=\frac{1}{2}[y\times d^{2}+\left(1-y\right)\times {\left(\max\;(m-d,0)\right)}^{2}]$$

where  y is a binary value, indicating whether the two inputs belong to the same class (if the two inputs belong to the same class, the value of y is set to one; otherwise it is set to zero); d is the Euclidean distance between the two extracted output feature vectors; and m is the margin value when the samples are dissimilar. The dissimilar input vectors only contribute to the loss function if their distance is smaller than the margin. The aim of training is to make the distance between the feature vectors of two similar inputs as small as possible and the distance between the feature vectors of two different inputs as large as possible.

After the training of the feature extraction model, the decision model is then trained with the feature vectors extracted using the feature extraction model. The loss function of the decision model is defined as follows:

(7) $${loss}_{d}=\frac{1}{2}{\left(y_{d}-y_{t}\right)}^{2}$$

where $y_{d}$ is the output of the decision model; $y_{t}$ is the true similarity of a given input in the training dataset, $y_{t}$ is set to one if the two feature vectors that formed the input belong to the same class, otherwise it is set to zero.

The BP network of the decision model has a three-layer architecture, i.e., an input layer with 64 neurons, a middle layer with 32 neurons, and an output layer with 1 neuron. The input is a 64-dimensional vector and the similarity of the two input vectors is calculated from the full-connection layer with 32 neurons. The similarity is a number between 0 and 1 and it will be compared with a pre-set threshold. When the similarity is larger than the threshold, the two inputs are considered to belong to the same class; otherwise, the two inputs are considered to belong to different classes.

When an unknown load is detected, the two BP networks of the feature extraction model and the decision model can be retrained to update the whole model.

## 4. Results

The Worldwide Household and Industry Transient Energy Dataset (WHITED) and the Plug Load Appliance Identification Dataset (PLAID) are used to benchmark the proposed method. Then, the feasibility of online training for BP networks on the embedded Linux system is validated. Finally, the proposed method is compared with other load identification methods.

### 4.1. Experiment Results Using the WHITED Dataset

#### 4.1.1. Siamese Network Pre-Training and Feature Database Construction

The WHITED dataset includes the voltage and current data of appliances sampled at 44 kHz, for 46 different appliance types. To mitigate the requirement on the sampling rate, the sampling rate can be reduced to 5.5 kHz under the condition that the V-I trajectory is not distorted. A complete V-I trajectory can be obtained when more than 100 samples are collected in one cycle and this requirement can be easily achieved using available sampling equipment [33]. For this, one sample is taken from every eight samples of the WHITED dataset.

In this paper, 30 different loads are used for verification, among which Load 1 to Load 18 are assumed to be known, while Load 19 to Load 30 are assumed to be unknown. The load names and labels are shown in Table 2 and the label represents the load in the subsequent analysis.

**Figure 4:**
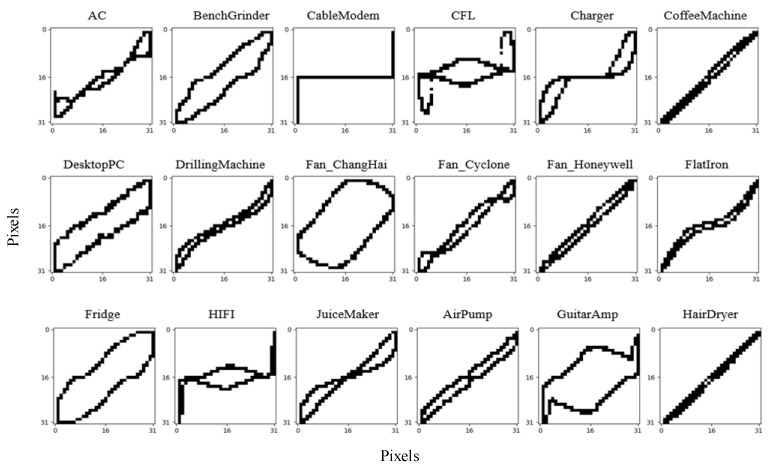
V-I trajectories of known loads (Load 1 to Load 18).

Figure 4 shows the V-I trajectories of Load 1 to Load 18. It can be seen that the V-I trajectories of Load 6 and Load 18 are very similar, so they are noted as being the same type. The Siamese network is trained with 17 different V-I trajectory images of 18 known loads and each image type contains 20 samples.

After the pre-training of the Siamese network, the feature database needs to be constructed, as it is a crucial foundation for the algorithm. The initial feature database is constructed with 17 V-I trajectory feature vectors extracted through the CNN network and the corresponding 18 power features of the 18 known loads from the WHITED dataset. It should be noted that, in the feature database, each V-I trajectory feature vector can correspond to the set of several load power features, because some loads have the same V-I trajectory, but different powers, as shown in Figure 1.

After the detection of an unknown load, the feature database will be dynamically updated. When the V-I trajectory of an unknown load is not in the feature database (i.e., the unknown load is identified using the Siamese network), both the V-I trajectory feature vector and its corresponding power feature need to be added into the feature database. When the V-I trajectory of the unknown load is the same as one of those in the feature database (i.e., the unknown load is identified using power feature matching), only the power feature is needed to be added into the feature database and needs to be mapped to the existing V-I trajectory vector.

#### 4.1.2. Retraining of the BP Networks

**Figure 5:**
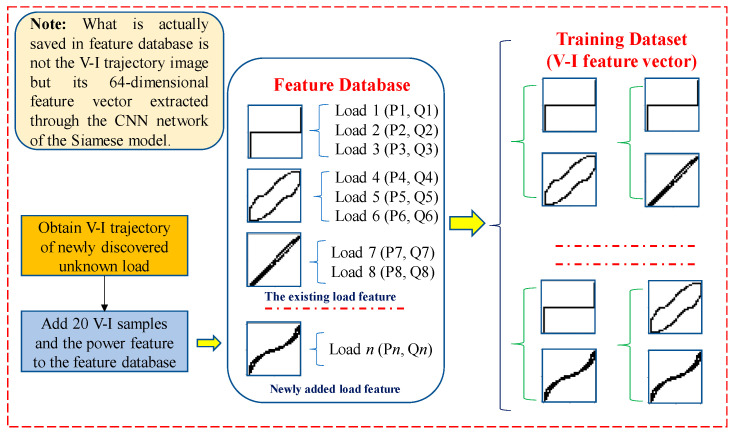
Example of constructing retraining dataset.

The BP networks will be retrained after an unknown load is found with a different V-I trajectory (for an unknown load found using power feature matching, retraining is not needed). In order to retrain the BP networks, the training dataset involving the V-I trajectory features must be reconstructed. The process of training dataset reconstruction is shown in Figure 5. It should be noted that, in the feature database, it is not the V-I trajectory image itself, but the 64-dimensional feature vector extracted from the V-I trajectory through the CNN network that is saved. Thus, as described in Section 3.2, the time required for identification can be greatly reduced.

Although, it is better when more samples are added to the feature database, as it will lead to a larger amount of calculation. Considering the need for retraining on the embedded terminal, 20 V-I trajectory samples obtained from the steady-state voltage and current data, as well as the corresponding power feature for one unknown load, are added to the feature database.

After updating the feature database, the training dataset will be reconstructed using the pairwise combination of the V-I trajectory feature vectors. Finally, only the BP networks in the feature extraction model and the decision model will be retrained.

#### 4.1.3. Identification Results

In order to verify the improvement effects after retraining, the identification accuracies both with and without retraining are compared. After several validations, the optimal similarity threshold of the Siamese model is found to be 0.8. When comparing the power features, the threshold value is set to 0.9.

In the experiment, Precision, Recall, and $F_{1}$-score are used as performance indicators, where Precision and Recall refer to the correct rate and recall rate, while the $F_{1}$-score indicates the harmonic mean of the Precision and Recall. Their calculation formulas are shown in (8)–(10), where TP, TN, FP, and FN refer to true-positive cases, true-negative cases, false-positive cases, and false-negative cases, respectively.

(8) $$Precision=\frac{TP}{TP+FP}$$

(9) $$Recall=\frac{TP}{TP+FN}$$

(10) $$F_{1}=2\cdot \frac{Precision\times Recall}{Precision+Recall}$$

The first step is to validate the identification accuracy for known loads. As the V-I trajectories of known loads are already used to train the Siamese networks, the identification accuracy of 18 known loads is up to 100%.

Then, the performance of unknown load detection is verified and the validation results are shown in Table 3. The outcomes illustrated that the proposed method could accurately detect the unknown loads. Among the unknown loads, the V-I trajectories of the Iron (Load 20) and the Network Switch (Load 28) have a high similarity with the known load of the Hair Dryer (Load 18) and the Cable Modem (Load 3), respectively. Therefore, they would be wrongly labeled as known loads, if only the V-I trajectory features were compared. However, with the help of further analysis based on power feature matching, Load 20 and Load 28 can be accurately classified into unknown loads. And then, their power features have to map to the corresponding V-I trajectory in the feature database.

**Figure 6:**
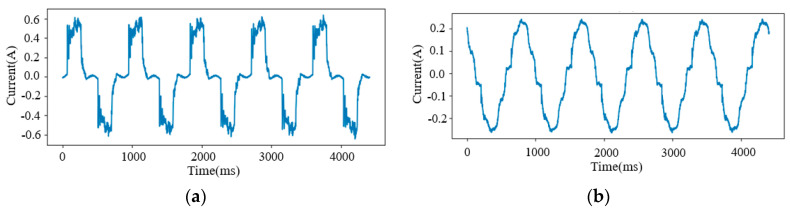
Current waveforms. (**a**) Load 29; (**b**) Load 21.

The validation results show that retraining the BP model can improve the identification accuracy of unknown loads. Because the Led Light (Load 21), Microwave (Load 22), Sewing Machine (Load 25), and Laptop (Load 29) fluctuate greatly during operation (the current waveforms of the Laptop and the Led Light are shown in Figure 6), the features extracted using the Siamese model are also unstable. Therefore, the BP network cannot offer a high-precision similarity. This issue can be solved by adjusting the parameters of each neuron in the BP model, which can prompt identification accuracy. The average $F_{1}$-score of 12 unknown loads has been improved from 0.9392 to 0.9917 with retraining.

### 4.2. Experiment Result using the PLAID Database

The PLAID dataset includes voltage and current data sampled at 30 kHz for 11 different appliance types captured in 55 households. The Siamese network was trained on the WHITED dataset and tested on the PLAID dataset, to verify the transferability.

**Figure 7:**
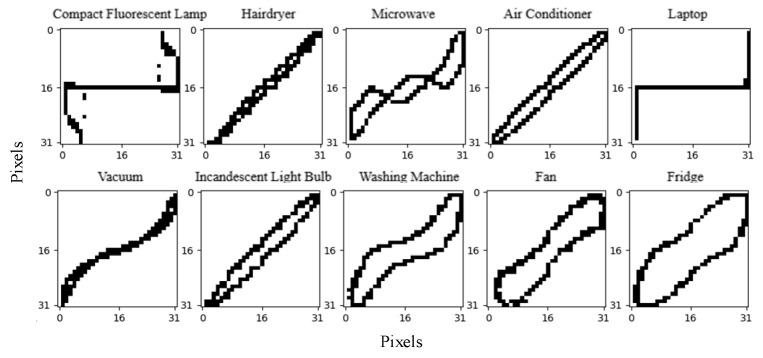
V-I trajectories of some loads in the PLAID dataset.

To verify the effectiveness of the proposed method, the experimental results of 10 different loads are presented and their V-I trajectories are shown in Figure 7. The initial feature database already has 18 known loads from the WHITED dataset and the identification results are shown in Table 4.

Since the V-I trajectories of the Laptop, Fridge, and Hairdryer of the PLAID dataset are the same as those of the Cable Modem, Fridge, and Coffee Machine of the WHITED dataset in the feature database, they can be detected as unknown loads using power feature matching and only the power features need to be added into the feature database. The V-I trajectories of other loads are different from those in the database; hence, they can be detected using the Siamese network. Then, the feature database is updated by adding both the V-I trajectory and its corresponding power feature, and the training dataset is reconstructed to retrain the two BP networks in the Siamese model.

The results illustrate that the proposed method can accurately identify unknown loads from different datasets and that the identification accuracy can be increased after retraining. The average $F_{1}$-score has been improved from 0.9483 to 0.9960.

### 4.3. Validation in the Real-House Environment Using the Embedded Linux System

#### 4.3.1. TensorFlow Lite

**Figure 8:**
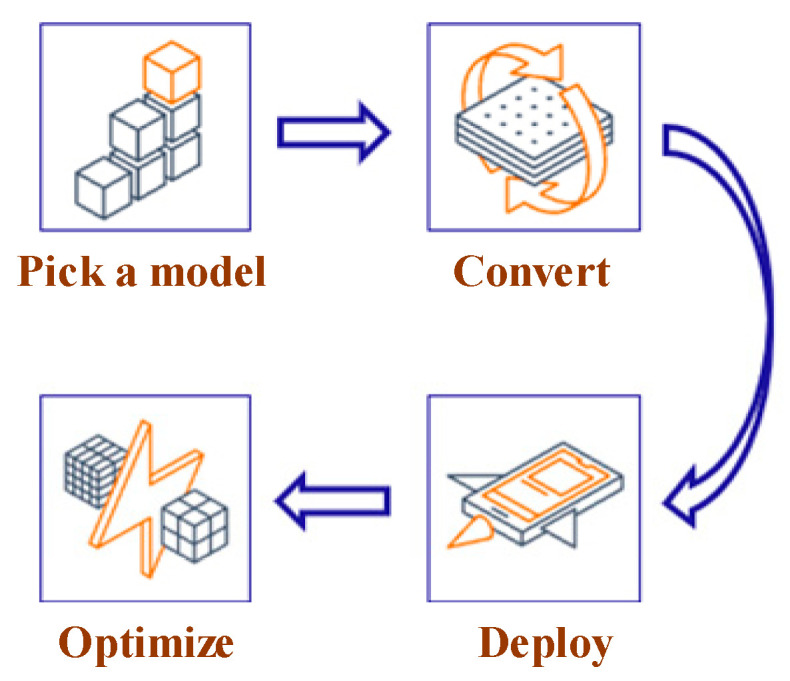
Model conversion using TensorFlow lite.

TensorFlow Lite is a framework that enables machine learning on mobile, embedded, and IoT devices. It can help to meet the real-time requirements of the NILM system and power consumption privacy. As shown in Figure 8, the workflow of TensorFlow Lite deployment is as follows:

- Model selection: Select a new model or retrain an existing one;
- Conversion: Convert a TensorFlow model into a compressed flat buffer through the TensorFlow Lite Converter;
- Deployment: Load the compressed “.tflite” file into a mobile or embedded device;
- Optimization: Quantize by converting 32-bit floats to more efficient 8-bit integers or run on GPU.

#### 4.3.2. Deployment of NILM Model

In this paper, the CNN network of the Siamese model is pre-trained on the computer and the TensorFlow Lite Converter compress this CNN model to a “.tflite” file, to load it into the embedded Linux system with STM32MP1 as the core. The STM32MP1 has dual cores—namely, A7, with a running frequency up to 800 MHz; and M4, with a running frequency up to 209 MHz. Any embedded device with a higher or similar performance can be used as the hardware platform. The size of the CNN network is approximately 80 KB after conversion and it takes around 20 ms to run this model on the embedded Linux system.

The two BP networks are implemented in the Python 3.7 environment, using the NumPy library on the embedded Linux system and each network is about 20 KB in size. It takes about 80 ms to complete the whole identification process, so the proposed method can meet the real-time requirements of the NILM system.

The initial feature database is in the form of csv files, which save the V-I trajectory feature vectors and the corresponding set of power features from Load 1 to Load 18 of the WHITED dataset.

Six different loads are included in the lab experiment, which are Microwave, Fridge, Heater, Hair Dryer, Laptop, and Iron. However, different operational states of the same device often exhibit distinct characteristics, making it challenging for the model to categorize them into the same class, such as is the case with the heater and the hairdryer. Therefore, we divided the multi-state device into different types of loads, such as Heater 1, Heater 2, Hairdryer 1, and Hairdryer 2, respectively. In addition, all loads are operated as an independent switched On/Off event, to acquire their voltage and current data.

**Figure 9:**
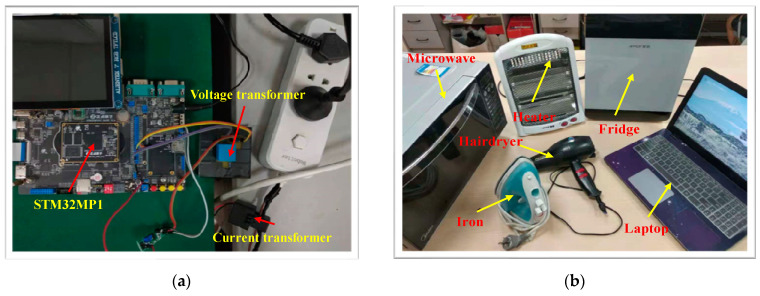
(**a**) NILM hardware system; (**b**) lab-loads used in validation.

The hardware for the NILM system, as well as the laboratory-based loads utilized for validation purposes, are depicted in Figure 9a and Figure 9b, respectively. The data acquisition is conducted at a frequency of 10 kHz. Throughout the experimental procedure, it is postulated that only a single load-switching event occurs at any given instance. The detection of these load-switching events is accomplished through the application of the Cumulative Sum Control Chart (CUSUM) algorithm [39], a prevalently employed methodology for identifying points of change.

**Figure 10:**
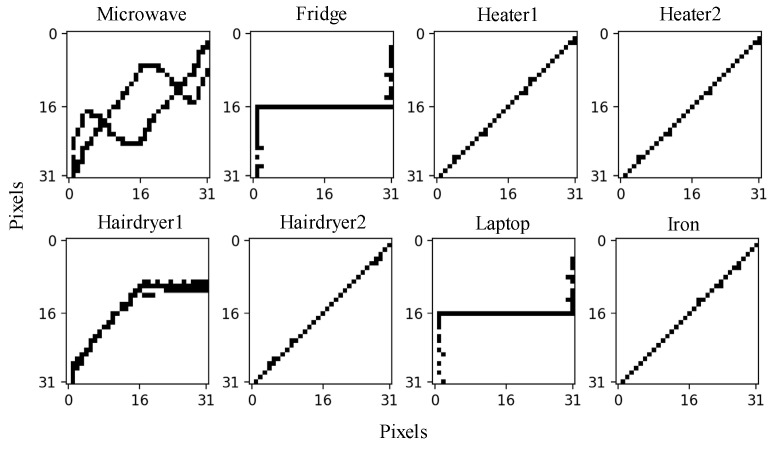
V-I trajectories of lab-loads.

After the system runs, all six loads in Figure 9b are identified as unknown loads using the proposed method; their V-I trajectories and power features are shown in Figure 10 and Table 5.

It should be noticed that the Heater and Hairdryer have two working modes. By comparing Figure 10 and Figure 4, it can be seen that the V-I trajectories of the Fridge and Laptop are similar to Load 3, and the V-I trajectories of Heater 1, Heater 2, Hairdryer 2, and the Iron are similar to Load 6; thus, these loads can be detected using power feature matching. But, the V-I trajectories of the Microwave and Hairdryer 1 are not in the feature database constructed from the WHITED dataset; hence, they can be detected through the Siamese network.

Since the V-I trajectories of the Microwave and Hairdryer1 did not participate in the pre-training, their identification accuracy is relatively low, at about 70%. Therefore, retraining is needed to enhance the identification accuracy. In total, 20 V-I trajectory vectors of the Microwave and Hairdryer1 are extracted using the CNN network, respectively, to be added to the feature database. The training dataset is reconstructed via the pairwise combination of the V-I trajectory feature vectors and the existing model is loaded to initialize the weights of the model. And then, the BP networks in the feature extraction model and the decision model are retrained on the embedded Linux system.

**Figure 11:**
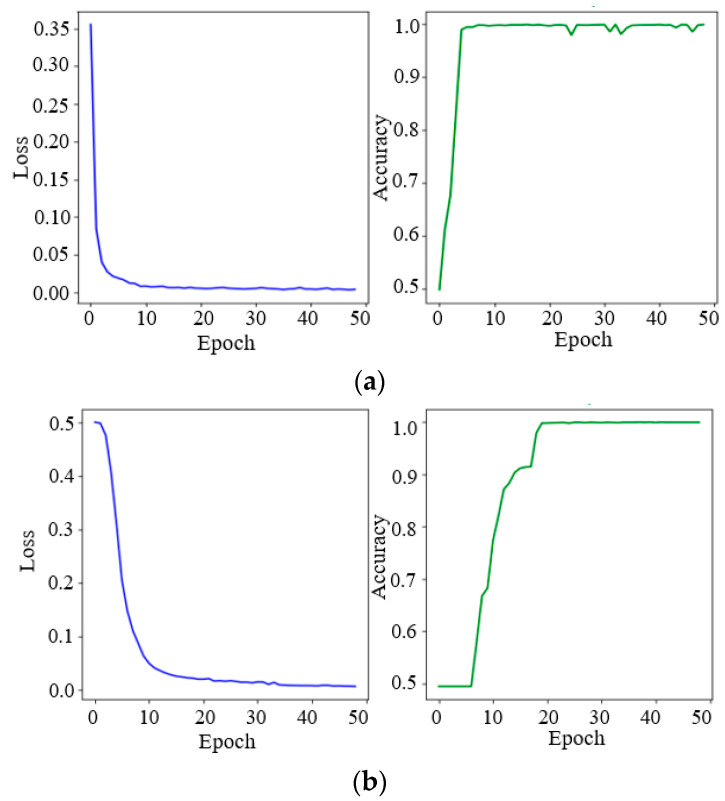
Online training process. (**a**) BP network in the feature extraction model; (**b**) BP network in the decision model.

During the BP network training process, the learning rate is set to 0.01. As shown in Figure 11, the loss value has converged to 0.1 after 10 iterations and the accuracy of the test dataset is up to 99.5%.

The identification results with and without retraining are shown in Table 6. It can be seen that the identification accuracy of the Microwave and Hairdryer1 has been considerably enhanced after retraining. The identification accuracy for the Laptop is not obviously enhanced after retraining, since the power of the Laptop is unstable. The identification accuracy of other loads with stable power is very high. The final average $F_{1}$-score of the lab-loads has been improved from 0.9124 to 0.9875, with retraining.

The experimental results show that 50 training iterations took 177 s, in total, when there are 19 different types of V-I trajectories (17 from the initial database and 2 from the Microwave and Hairdryer1) in the training set. It can be demonstrated that the proposed method can achieve the real-time online training function on the embedded Linux system, to enhance the identification accuracy and this is the foundation of the scalability for the NILM system.

### 4.4. Comparison with Other Algorithms

The proposed method is compared with others to validate the performance through four technical indicators, i.e., ACC, $F_{macro}$, unknown load detection ability, and deployment difficulty. ACC is the ratio of the correctly identified observations to the total observations, and $F_{macro}$ refers to the macro-averaged $F_{1}$ score, which is calculated by taking the average of the $F_{1}$ scores of each class in the dataset. The calculation formulas of ACC and $F_{macro}$ are shown in (11) and (12).

(11) $$ACC=\frac{TP+TN}{TP+TN+FP+FN}$$

(12) $$F_{macro}=\frac{1}{N}\sum_{i=1}^{N}{F1}_{i}$$

where *N* is the number of classes and ${F_{1}}_{i}$ is the $F_{1}$-score for class *i*. In addition, the unknown load detection ability determines whether the algorithms are capable of detecting unknown loads and the deployment difficulty is evaluated by the number of required devices and computation dependence in actual applications. For example, the deployment difficulty is considered high when a PC or Server for computing support and communication devices is required during real-time operation, while the deployment difficulty is considered low when no additional devices are required. The comparison results are shown in Table 7.

In [21], only the binary V-I trajectory is used as the load feature; thus, the identification accuracy is not high, because the V-I trajectories of loads with the same internal circuit are similar. In [3], the identification accuracy is increased by using color-encoding techniques. The load identification is defined as a multi-classification issue in [3,21], which only applies to the known item classification. Hence, both approaches cannot detect unknown loads and the classification model has to be retrained, as a whole, to serve the new load.

Both methods in [29,31] can identify unknown loads. However, the model complexity is high and the system requires high computing power, which brings a considerable barrier when it comes to deployment on the embedded system. Although the methods in [30,33] can be implemented on the embedded system, the Siamese network model and the autoencoder model for feature extraction are fixed. Thus, the accuracy of the NILM system will be affected by the increased number of unknown loads.

The proposed method in this paper can accurately detect unknown loads by calculating the similarity of the V-I trajectory and power feature matching. Furthermore, with the number of unknown loads increasing, the model can be retrained online on the embedded systems. From the comparison results, it can be seen that the proposed method is more scalable than state-of-the-art methods

## 5. Conclusions and Future Research Directions

This paper has proposed a non-intrusive load identification method based on a retrainable Siamese network that is composed of a fixed CNN network and two retrainable BP networks. The CNN network and one of the BP networks extract the low-dimensional feature vectors of the V-I trajectories, while the other BP network calculates the similarity of the feature vectors. According to the similarity, whether the load V-I trajectory is identified as unknown or known will be determined. If the load V-I trajectory is identified as unknown, the BP networks can be subsequently retrained, so that the performance of the Siamese network will be improved. If the load V-I trajectory is identified as known, power feature matching is executed.

The proposed method can be implemented on an embedded Linux system with online retraining. Therefore, it can improve the real-time performance of the NILM system and the privacy of the customer. The WHITED dataset and the PLAID dataset are used to verify the performance of the proposed method. Finally, the practicality and scalability are validated using the real-house environment test to prove the ability of online retraining on an embedded Linux system with STM32MP1 as the core. From the experimental results, the proposed method can be efficiently generalized, compared with state-of-the-art methods.

However, there are still some limitations of the proposed method. For instance, the method does not identify the simultaneous switching events on multiple appliances. Also, the switching events in which a single load has various working states are not discussed. Subsequent research will focus on solving these issues.

The load identification results of our research have paved the way for efficient energy management. With the individual load consumption information, the users can know their energy consumption status in real time; thus, they can adjust their behaviors to save energy. In addition, the load information can be input into the Home Energy Management Systems (HEMS), which can automatically move the shiftable and interruptible loads to the valley period to reduce the expenses [40,41]. Moreover, the HEMS can participate in the demand-side response with the knowledge of load consumption information. In the future, we will incorporate the NILM with the HEMS for smart energy management.

## Figures and Tables

**Table 1:** Comparison between different NILM methods.

Ref.	Feature	Model	Real-Time Operation	Unknown Load Detection	Model Real-Time Updating	Computing Support from PC or Server in Operation
[10]	Power	HMM	Disable	Disable	Disable	Necessary
[14]	Power	Seq2point	Disable	Disable	Disable	Necessary
[13]	Weighted V-I image	CNN	Enable	Disable	Disable	Unnecessary
[16]	Reconstructed V-I image	CNN	Enable	Disable	Disable	Unnecessary
[3]	Colored V-I image	AlexNet	Enable	Disable	Disable	Necessary
[22]	Binary V-I image	Siamese Model	Enable	Enable	Disable	Unnecessary
[23]	Binary V-I image + Power	Siamese Model	Enable	Enable	Disable	Necessary
[25]	Binary V-I image + FFT	Autoencoder + TOPSIS	Enable	Enable	Disable	Unnecessary
[21]	Current	1D-LeNet Siamese Model	Enable	Enable	Enable	Necessary
**Proposed**	**Binary V-I image + Power**	**Retrainable Siamese Model**	**Enable**	**Enable**	**Enable**	**Unnecessary**

**Table 2:** Loads from the WHITED datasets used in validation.

Label	Name	(P, Q)	Label	Name	(P, Q)
Load 1	AC	(330, 43)	Load 16	Air Pump	(100, 18)
Load 2	Bench Grinder	(370, 140)	Load 17	Guitar Amp	(17, 20)
Load 3	Cable Modem	(4, 2)	Load 18	Hair Dryer	(1940, 135)
Load 4	CFL	(13, 2)	Load 19	Kitchen Hood	(110, 155)
Load 5	Charger	(70, 17)	Load 20	Iron	(1430, 110)
Load 6	Coffee Machine	(790, 65)	Load 21	Led Light	(35, 11)
Load 7	Desktop PC	(100, 45)	Load 22	Microwave	(1340, 270)
Load 8	Drilling Machine	(310, 45)	Load 23	Monitor	(55, 18)
Load 9	Fan_ChingHai	(25, 40)	Load 24	Power Supply	(12, 15)
Load 10	Fan_Cyclone	(280, 42)	Load 25	Sewing Machine	(150, 60)
Load 11	Fan_Honeywell	(136, 15)	Load 26	Vacuum Cleaner	(705, 60)
Load 12	Flat Iron	(280, 30)	Load 27	Rice Cooker	(330, 7)
Load 13	Fridge	(560, 285)	Load 28	Network Switch	(2, 0.5)
Load 14	HIFI	(29, 17)	Load 29	Laptop	(67, 20)
Load 15	Juice Maker	(220, 45)	Load 30	Water Pump	(450, 75)

**Table 3:** Identification results using the WHITED dataset.

Label	Unknown Load Detection	Without Retraining			With Retraining		
		Precision	Recall	F_1_-Score	Precision	Recall	F_1_-Score
Load 19	100%	100.00%	100.00%	1.0000	100.00%	100.00%	1.0000
Load 20	100%	100.00%	100.00%	1.0000	100.00%	100.00%	1.0000
Load 21	100%	85.00%	68.00%	0.7556	97.96%	96.00%	0.9697
Load 22	100%	100.00%	78.00%	0.8764	100.00%	96.00%	0.9796
Load 23	100%	100.00%	100.00%	1.0000	100.00%	100.00%	1.0000
Load 24	100%	100.00%	100.00%	1.0000	100.00%	100.00%	1.0000
Load 25	100%	73.33%	88.00%	0.8000	96.08%	98.00%	0.9703
Load 26	100%	100.00%	100.00%	1.0000	100.00%	100.00%	1.0000
Load 27	100%	100.00%	100.00%	1.0000	100.00%	100.00%	1.0000
Load 28	100%	100.00%	96.00%	0.9796	100.00%	100.00%	1.0000
Load 29	100%	100.00%	92.00%	0.9583	100.00%	100.00%	1.0000
Load 30	100%	81.97%	100.00%	0.9009	96.15%	100.00%	0.9804

**Table 4:** Identification results using the PLAID dataset.

Name	Without Retraining			With Retraining		
	Precision	Recall	F_1_-Score	Precision	Recall	F_1_-Score
Compact Fluorescent Lamp	95.92%	94.00%	0.9495	98.04%	100.00%	0.9901
Hairdryer	100.00%	98.00%	0.9899	100.00%	98.00%	0.9899
Microwave	100.00%	100.00%	1.0000	100.00%	100.00%	1.0000
Air Conditioner	98.04%	100.00%	0.9901	100.00%	100.00%	1.0000
Laptop	94.12%	96.00%	0.9505	100.00%	98.00%	0.9899
Vacuum	100.00%	100.00%	1.0000	100.00%	100.00%	1.0000
Incandescent Light Bulb	98.00%	98.00%	0.9800	98.04%	100.00%	0.9901
Washing Machine	86.67%	78.00%	0.8211	100.00%	100.00%	1.0000
Fan	100.00%	96.00%	0.9796	100.00%	100.00%	1.0000
Fridge	77.19%	88.00%	0.8224	100.00%	100.00%	1.0000

**Table 5:** Power features of the lab-loads.

Name	Active Power (W)	Reactive Power (Var)
Microwave	566	100
Fridge	30	10
Heater1	156	5
Heater2	304	12
Hairdryer1	156	5
Hairdryer2	200	5
Laptop	16	10
Iron	605	14

**Table 6:** Identification results on lab-loads.

Name	Without Retraining			With Retraining		
	Precision	Recall	F_1_-Score	Precision	Recall	F_1_-Score
Microwave	100.00%	70.00%	0.8235	100.00%	100.00%	1.0000
Fridge	90.57%	96.00%	0.9320	92.45%	98.00%	0.9515
Heater1	80.00%	100.00%	0.8889	100.00%	100.00%	1.0000
Heater2	100.00%	100.00%	1.0000	100.00%	100.00%	1.0000
Hairdryer1	100.00%	75.00%	0.8571	100.00%	100.00%	1.0000
Hairdryer2	100.00%	100.00%	1.0000	100.00%	100.00%	1.0000
Laptop	95.74%	90.00%	0.9278	97.87%	92.00%	0.9485
Iron	76.92%	100.00%	0.8696	100.00%	100.00%	1.0000

**Table 7:** Comparison of different algorithms using the PLAID dataset.

Ref.	Feature	Model	Dataset	Accuracy (%)	$F_{macro}$	Unknown Load Detection	Deployment Difficulty
[21]	Binary V-I image	CNN	All loads in PLAID	78.50	0.7760	Disable	Easy
[3]	Colored V-I image	AlexNet	All loads in PLAID	98.04	0.9540	Disable	Difficult
[31]	Binary V-I image + Power	Siamese Model	House6 in PLAID	**/**	0.9788	Enable	Difficult
[29]	Current	1D-LeNet Siamese Model	6 loads in PLAID	99.80	**/**	Enable	Difficult
[30]	Binary V-I image	Siamese Model	11 loads in PLAID	99.40	0.8990	Enable	Easy
[33]	Binary V-I image + FFT	Autoencoder + TOPSIS	11 loads in PLAID	97.60	**/**	Enable	Easy
Pro-posed	Binary V-I + Power	Retrainable Siamese Model	10 loads in PLAID	99.60	0.9920	Enable	Easy

## Data Availability

Data will be made available on request.
